# Enzymatic synthesis of geranyl acetate in packed bed reactor in supercritical carbon dioxide under various pressure-temperature conditions and reactor configurations

**DOI:** 10.1016/j.dib.2018.07.010

**Published:** 2018-07-20

**Authors:** Mohamed Chafik Bourkaib, Harifeno Randriamalala, Lena Dettori, Catherine Humeau, Stephane Delaunay, Isabelle Chevalot, Yann Guiavarc'h

**Affiliations:** Laboratory Reactions and Process Engineering, University of Lorraine, CNRS, LRGP, F-54000 Nancy, France

## Abstract

Data in this paper describes the catalytic performances, expressed in terms of conversion %, of geraniol and acetic acid to geranyl acetate, using the immobilized lipase B from *Candida antarctica* in packed bed reactors (PBR) using supercritical CO_2_ as a solvent. Readers will find data related to different Figures or equations of the article as well as supplementary data that will help to make the difference between flowrates of CO_2_ in a liquid state and corresponding flowrates of supercritical CO_2_ for various CO_2_ pressure and temperature combinations.

**Specifications Table**

**Table:** 

Subject area	*Process biochemistry*
More specific subject area	*Green catalytic processing in supercritical CO*_*2*_
Type of data	*Table, figure*
How data was acquired	*Immobilized enzyme water activity was checked to be 0.23 with a Novasina Lab-Master aw-meter.*
	*Data on enzyme activity were acquired using an Agilent 7890A gas chromatograph equipped with a FID detector for quantification and coupled with an Agilent 7000A MS triple quad detector (Agilent technologies, Santa Clara, USA). An internal standard was used.*
Data format	*Raw, analyzed*
Experimental factors	*CO*_*2*_*pressure in the range 100–300 bar, CO*_*2*_*temperature in the range 45–65* *°C, CO*_*2*_*Flow rate in the range 0.5 to 6* *mL/min, packed-bed reactor volume (1* mL, 2*1 *mL in parallel, or 5* *mL), reaction time (0–22 hours), reaction cycles (6)*
Experimental features	– *study of the effect of pressure and temperature of supercritical CO*_*2*_ *on the enzymatic synthesis of geranyl acetate using a full factorial Design of Experiment with central point.* – *study of time at which steady-state conversion was achieved for short and long PBR.* – *study of catalytic stability in the PBR over a long reaction time or several repeated runs with depressurization-repressurization.* – *study of the impact of CO*_*2*_ *flow-rate (i.e. residence time) in the PBR on the conversion % at 55* *°C-200 bar and 65* °*C–150 bar*
Data source location	*/*
Data accessibility	*Data is with this article*
Related research article	*Enzymatic synthesis of geranyl acetate in packed bed reactor in supercritical carbon dioxide under various pressure-temperature conditions and reactor configurations “in press.”*

**Value of the data**

• These data describe, for the first time, the combined effect of pressure and temperature variations of supercritical CO_2_ on *Candida antarctica* lipase B (CALB) catalytic properties.
• These data show how important is the appropriate control of CO_2_ flow-rate (i.e. reaction mixture residence time) for the achievement of a very high conversion % together with a good process productivity.
• These data may be considered with interest by any immobilized enzyme suppliers as well as manufacturers interested in continuous, green and enzymatic synthesis of terpenes esters.

## 1 Data

The collection of the experimental data in this paper aimed to contribute to the promotion of supercritical CO_2_ as a green, cheap, safe solvent for the synthesis of geranyl acetate, a widely used terpene ester. In this data in brief article, we found appropriate to focus on data related to the design of experiments on the influence of pressure and temperature on the conversion % of geraniol to geranyl acetate. We then present data related to the study of the impact of the supercritical CO_2_ residence time in the packed bed reactor (PBR) on the conversion %. Finally we give supplementary data helping the reader to easily get the densities, flow rates and residence times of various supercritical CO_2_ produced from a -8°C liquid CO_2_ pumped at various flow-rates. This last point is also important since when CO_2_ expand when passing from the liquid state to the supercritical state, its density, flow-rates but also the substrates concentrations in it, change.

## 2 Experimental design, materials, and methods

### 2.1 Design of Experiments (DoE)

Table 1 below presents the pressure-temperature conditions used in our design of experiment as well as the corresponding conversion % of geraniol to geranyl acetate.

**Table 1:** Pressure and temperature conditions of the design of experiment.

**code**	**Pressure (bar)**	**Temperature (°C)**	**Conversion %**
−−	150	45	27,24
−−	150	45	29,88
−−	150	45	32,73
−+	150	65	74,84
−+	150	65	73,23
−+	150	65	73,18
0	225	55	42,93
0	225	55	45,05
0	225	55	46,21
0	225	55	44,43
0	225	55	44,01
0	225	55	47,61
+−	300	45	26,51
+−	300	45	28,06
+−	300	45	29,28
++	300	65	58,58
++	300	65	57,93
++	300	65	59,74

Pressure was in the range 150–300 bars with a central point at 225 bar. Temperature was in the range 45–65 °C with a central point at 55 °C (− and + indicate the low and high level, respectively, for each factor. 0 indicate a central level for each factor). For each P-T conditions, a flow rate of 3 mL min^−1^ of liquid CO_2_ at −8 °C was pumped, converted to supercritical CO_2_ at the target pressure and temperature, and mixed with of an equimolar mixture of acetic acid and geraniol pumped at flow rate of 40 µL min^−1^ prior to pass through a 1 mL PBR filled with 300 mg of lipozyme®435 equilibrated at a water activity of 0.23 (water activity measurement performed with a Novasina Lab-Master aw meter). The substrates concentration in CO_2_ was therefore 57 mM, based on the CO_2_ flow in a liquid form (i.e. prior to density changes when increasing pressure and temperature). Unreacted substrates as well as products geranyl acetate (no visible water traces), were collected in a liquid form by CO_2_ depressurization through a back-pressure regulator-separator and a CO_2_ flow-breaker. Collecting vials were 60 mL amber vials. The run time was 30 min.

After each run, the collected volume was measured and 200 µL of this volume was added to 800 µL of acetone prior addition of 50 µL of methyl decanoate as an internal standard. After mixing, 1 µL of the sample was injected with a split ratio of 100:1 in a Agilent 7890 A gas chromatograph equipped with a Agilent HP-INNOWax 30 m × 0.32 mm internal diameter x 0.25 µm column and a FID detector for quantification. The GC-FID was also coupled with an Agilent 7000 A MS triple quad detector (Agilent technologies, Santa Clara, USA). Details are given in the article. The detection and quantification limits (LOD and LOQ) were determined for all standards according to a US-EPA method [1]. LOD and LOQ were calculated as [*3 × s*_*a0*_ */a*] and [*10 × s*_*a0*_ */a*] respectively, with *s*_*a0*_ and *a,* the standard deviation on the intercept and the slope of the calibration curve, respectively.

The conversion % was calculated according to equation 1 as the ratio between the amount of geranyl acetate produced (mole) and the amount of geraniol (mole) pumped during the run:

$$\mathrm{conversion}\;\%\;=\frac{\mathrm{amount}\;\left(\mathrm{mole}\right)\;\mathrm{of\backslash geranyl\backslash acetate\backslash produced}\;\mathrm{in}\;30\;\min\;\mathrm{run}}{\mathrm{amount}\;\left(\mathrm{mole}\right)\;\mathrm{of\backslash geraniol\backslash pumped\backslash in}\;30\;\min\;\mathrm{run}}\times 100$$

The four corner points conditions of the DoE were tested three times whereas the central point condition was tested six times, according to JMP 10 software from SAS institute Inc. (Cary, NC, USA) recommendations [2]. Fitting of these experimental data, in a coded form, could be achieved with JMP 10 based on a simple linear model with interaction between Pressure and Temperature (results can be seen in Table 1 of the article).

**Fig. 4:**
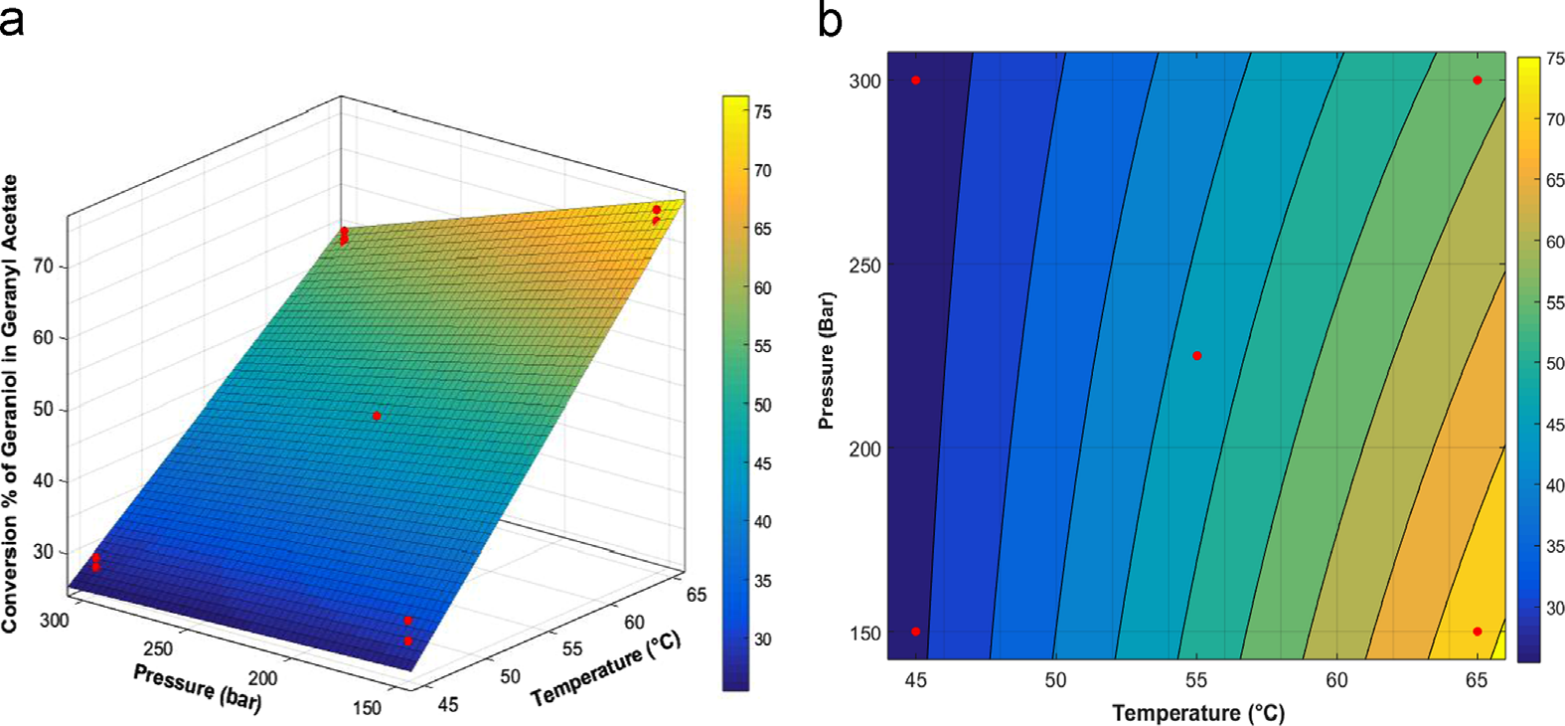
Surface response (a) and isoresponse curves (b) of geraniol conversion rate to geranyl acetate versus temperature and pressure of supercritical CO_2_ (similar conditions with Figure 3). Red dot are experimental points (3 at each corner and 6 in the central position).

In order to generate the surface response and isocontour plots corresponding to Fig. 4a and b of the article, respectively, we needed the coefficient of the model in a non-coded form. To do so we used the curve-fitting tool of Matlab (Mathworks, Natick, USA) and a non-linear regression based on the Levenberg-Marquardt algorithm (coefficients of the surface response equation can be seen in equation 2 of the article).

### 2.2 Study of the impact of supercritical CO_2_ (i.e. substrates) residence time in the PBR on the conversion % of geraniol to geranyl acetate

In our Design of experiment and stability tests, we used 3 mL/min of liquid CO_2_ pumped at 60 bar and −8 °C with a density of 0.991. All along the article, for convenience, we used the liquid CO_2_ flow-rate as a constant basis for substrates concentration, residence times and enzymatic conversion comparisons. But it is obvious that, when passing from liquid to supercritical, CO_2_ density, and therefore CO_2_ flow-rate and substrate concentrations in the supercritical CO_2,_ change. We used densities of supercritical CO_2_ as found with the Span and Wagner Equation of State defined in 1996. A free computational tool from the Energy Institute of the University of Pennsylvania was used to calculate the densities with this equation [3]. At 55 °C and 200 bar, the density of supercritical CO_2_ was 754 kg m^−3^. At 65 °C and 150 bar, this density was 0.559 kg m^−3^.

**Fig. 8:**
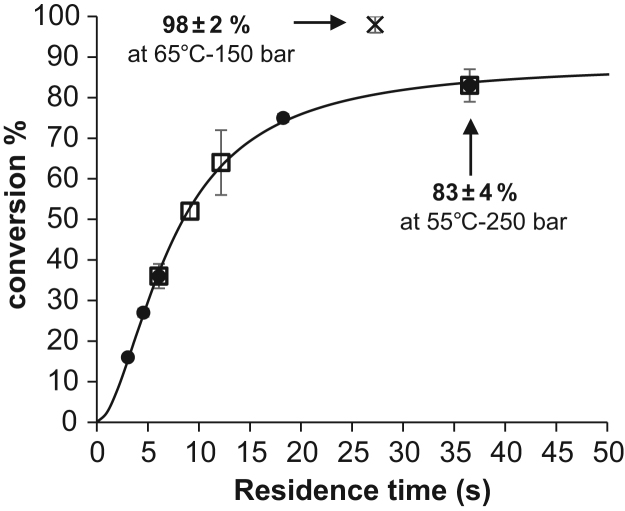
Influence of supercritical CO_2_ residence time (i.e. of scCO_2_ flow-rate) on the conversion rate of geraniol to geranyl acetate. Here, changes in CO_2_ density when passing from liquid state to supercritical state have been used to calculate the real flow rates and corresponding residence times of supercritical CO_2,_ as explained in supplementary material. (•) represent runs carried-out in one single short PBR containing 300 mg Lipozyme®435 beads, (⎕) represent runs carried-out in two short PBR in parallel (i.e. using 2×300=600 mg Lipozyme®435 beads). Experimental conditions: supercritical CO_2_ at 55 °C and 200 bar. Geraniol and acetic acid concentrations were kept to 57 mM based on the liquid CO_2_ pumped. Additional x marker represents runs carried-out in one single PBR containing 300 mg Lipozyme®435 beads with a supercritical CO_2_ at 65 °C and 150 bar at 27 s residence time (0.5 mL/min liquid CO_2_) with 57 mM of each substrates too, based on the liquid CO_2_ pumped. Runs were performed in triplicates.

Table 2, Table 3 below shows flow-rates for liquid and supercritical CO_2_ as well as the residence time of supercritical CO_2_ in the PBR, expressed in seconds, assuming that spaces between beads is about 40% as currently considered with spherical catalyst particles. Conversion % and corresponding standard deviations were calculated with n=3. These data were used to generate the Fig. 8 of the article on the impact of residence time of supercritical CO2 (and therefore reaction mixture) on the conversion %.

**Table 2:** Detailed data for CO_2_ flow rates, liquid and supercritical, residence time of supercritical CO_2_ in the PBR, and corresponding conversion % with their associated standard deviation. Experimental conditions: liquid CO_2_ pumped at −8 °C and 60 bar and converted to supercritical CO_2_ at 55 °C and 200 bar. Use of one single 1 mL PBR or of two 1 mL PBR in parallel. PBR were filled with 300 mg of Lipozyme®435 beads equilibrated at aw=0.23. Runs were performed in triplicate.

**nb PBR**	**liquid CO**_**2**_**flow (mL min**^**−1**^**)**	**supercritical CO**_**2**_**flow (mL min**^**−1**^**)**	**Residence time of supercritical CO**_**2**_**in PBR (seconds)**	**Conv. %**	**stdev conv %**
1	0.5	0.66	36.5	83	4
1	1.0	1.31	18.3	75	1
1	3.0	3.94	6.1	36	3
1	4.0	5.26	4.6	27	1
1	6.0	7.89	3.0	16	0
2	1	0.66	36.5	83	4
2	3	1.97	12.2	64	8
2	4	2.63	9.1	52	2
2	6	3.94	6.1	36	3

**Table 3:** similar data as compared to Table 2 but using supercritical CO_2_ at 65 °C and 150 bar and one 1 mL PBR. This point was added to Fig. 8 of the article.

**nb PBR**	**liquid CO**_**2**_**flow (mL min**^**−1**^**)**	**supercritical CO**_**2**_**flow (mL min**^**−1**^**)**	**Residence time of supercritical CO**_**2**_**in PBR (seconds)**	**Conv. %**	**stdev conv %**
1	0.5	0.88	27.3	98	2

Table 2 lists the liquid CO_2_ flow-rates (at −8 °C and 60 bar) used in experiments with one single short 1 mL PBR filled with 300 mg of Lipozyme®435 beads or with two short 1 mL PBR in parallel, also filled with 300 mg of Lipozyme®435 beads (300 mg/PBR). The third column shows the corresponding flow-rates once that the CO_2_ expanded when reaching the supercritical zone at 55 °C and 200 bar. These flow-rates are logically higher due to lower CO_2_ density. The fourth column gives the residence time of supercritical CO_2_ in the PBR (expressed in seconds).

Table 3 lists similar data as compared to Table 2 but using supercritical CO_2_ at 65 °C and 150 bar, the best experimental temperature and pressure found based on our Design of Experiment.
